# Rehabilitation and return to sports after isolated meniscal repairs: a new evidence-based protocol

**DOI:** 10.1186/s40634-022-00521-8

**Published:** 2022-08-17

**Authors:** Filippo Calanna, Victoria Duthon, Jacques Menetrey

**Affiliations:** 1 Centre de Médecine du Sport Et de L’Exercice, Swiss Olympic Medical Center, Hirslanden Clinique La Colline, Geneva, Switzerland; 2 1^ Clinica Ortopedica, ASST Centro Specialistico Ortopedico Traumatologico Gaetano Pini-CTO (Milan, Italy), Piazza Cardinal Ferrari 1, 20122 Milano, Italy; 3 Orthopaedic Surgery Service, University Hospital of Geneva, Geneva, Switzerland

**Keywords:** Meniscus, Meniscal suture, Traumatic tears, Rehabilitation protocol, RTS

## Abstract

**Purpose:**

Despite many protocols that have been proposed, there’s no consensus in the literature regarding the optimal rehabilitation program and return to sports (RTS) protocol following isolated meniscal repair. The aim of this current concept review is to look at the evidence of rehabilitation and RTS program after isolated meniscal repair, focusing on general and specific protocols per type of injury trying to give some guidelines based on the current state of knowledge.

**Methods:**

A narrative literature review was performed searching PubMed database to identify relevant articles from January 1985 to October 2021 on rehabilitation and RTS after isolated meniscal repair. Randomized controlled trials (RCTs), prospective and retrospective cohort studies, case series, systematic reviews, meta-analyses, cadaveric studies and basic science studies were included.

**Results:**

When the hoop tensile stress effect is preserved, an accelerated rehabilitation program may be suggested. Hence, partial weight bearing (20 kg) in association with ROM limited to 90° is allowed for the first four weeks, followed by weight bearing as tolerated.

In contrast, when circumferential hoop fibers are disrupted, a restricted rehabilitation protocol may be recommended. In this scenario no weight bearing is allowed for the first six weeks after the surgery and range of motion (ROM) is limited to 90°.

**Conclusion:**

Biomechanical evidence suggests that tailoring an individualized protocol based upon the type of lesion and meniscus stability is reasonable.

**Level of evidence:**

Level V.

## Introduction

Classification of meniscus tears is a very important tool for the assessment and treatment of the meniscal lesions.

In 2006, the International Society of Arthroscopy, Knee Surgery and ISAKOS Knee Committee developed an arthroscopically assessed classification of meniscal tears based on tear depth, location, tear pattern, length, tissue quality and percentage of the meniscus excised, that provided acceptable interobserver reliability [1]. Various “classical” tear patterns and configurations have been described and include the following; radial tear, longitudinal vertical/horizontal cleavage, flap or parrot-beak tear, bucket-handle lesion and complex tear [1].

Beside aforementioned “classical” meniscal lesions, new meniscal tears entities have been recently described and can be the cause of residual pain, mechanical symptoms and residual anteroposterior laxity [2, 3]. These meniscal lesions are encountered in menisco-synovial tear on the posterior horn of the medial meniscus, partial or complete posterior root tear of the medial or lateral meniscus, and the hypermobile lateral meniscus, associated to rotational laxity (Table 1) [2, 3].

**Table 1:** Meniscal tears classification

Meniscal tear	Description
Longitudinal Vertical	Vertically oriented parallel to the edge of the meniscus
Longitudinal Horizontal	Horizontally oriented perpendicular to the edge of the meniscus. The superior and the inferior surfaces of the meniscus are divided
Radial	Vertically oriented extending from the inner edge of the meniscus toward its periphery
Bucket Handle	The inner fragment of a longitudinal tear displaces over into the intercondylar notch
Flap or Parrot-Beak (oblique tear)	Radial tears with a circumferential extension creating a flap of meniscal tissue
Complex	Combination of other tears that occurred in multiple planes
Ramp (menisco-synovial)	Tears located at the posterior meniscocapsular junction and/or tears of the posterior meniscotibial ligament
Root	Defined as either radial/oblique tears located within 1 cm of the meniscal attachment or a bony/soft-tissue root avulsion
Hypermobile Lateral Meniscus	Hypermobile lateral menisci are thought to result from either congenital absence of posterior capsular attachments or from tears of posterior capsular attachment, in particular the popliteomeniscal fascicles

From a biomechanical point of view, the menisci have different functions; load distribution, shock absorption, cartilage nutrition, stability and the capacity of lower friction increasing the congruency of the joint [4].

**Fig. 1:**
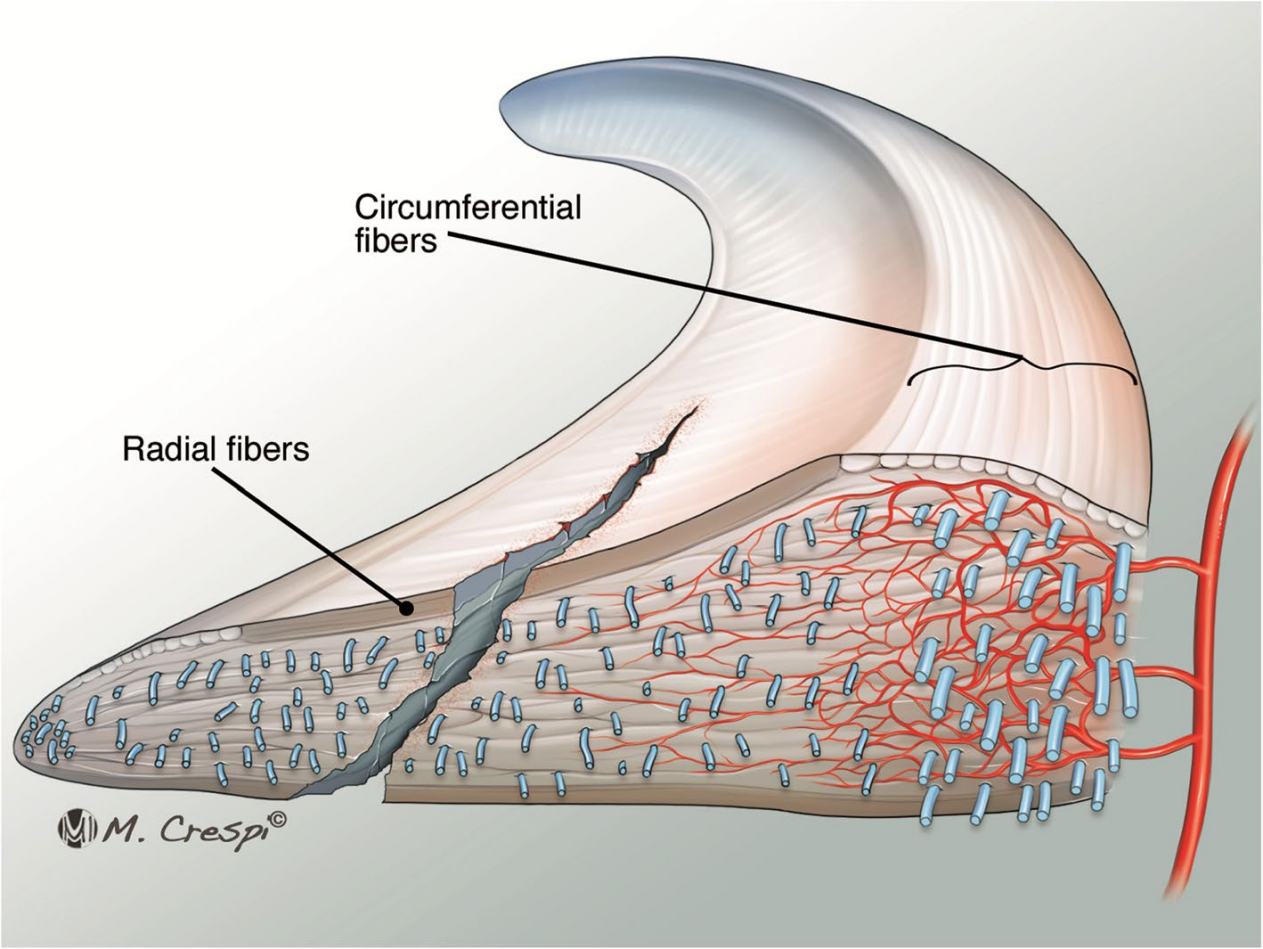
The meniscal micro-structure with two different orientations of the collagen fibers. The circumferential fibers, creating the so called “hoop stress effect”, and the radial ones, keeping the tissue-structure integrity. In case of vertical longitudinal tears scenario the hoop tensile stress effect is preserved and the circumferential fibers are intact. Differently from that, in case of radial lesion the circumferential fibers are disrupts and the hoop stress effect is dissolved

The load distribution and shock absorption effects are influenced by their macro-geometry and tissue-architecture. The menisci are basically constituted of interlacing networks of collagen fibers (mainly type I collagen) interposed among cells and an extracellular matrix (ECM) of glycoproteins and proteoglycans [4]. The menisci have a high percentage of water content (72%), giving the biomechanical function to resist compression and axial loading. The remaining 28% is composed of organic matter, mostly ECM and cells (fibrocytes, fibroblasts, meniscus cells, fibrochondrocytes, and chondrocytes) [4]. Concerning the micro-structure of the tissue, the meniscus has two different orientations of the collagen fibers. The circumferential fibers predominantly convert and disperse axial and compressive loading creating the so called “hoop stress effect”. Differently, the radial ones, have an important function to counter-attack longitudinal splitting forces of the circumferential collagen bundles, keeping the ultrastructure integrity (Fig. 1) [4]. Lastly, it has been advised that the inner third of the meniscus may play a major role in dealing with compression forces while the outer two-thirds counteract radial tension forces [4]. The collagen bundles included in the more superficial layer of menisci have a random orientation that somewhat mimics hyaline cartilage [4].

The consequences of these forces across the repair can vary depending on the tear type and location [5]. For example, in case of vertical longitudinal tears scenario where the hoop tensile stress effect is preserved and the circumferential fibers are intact, a compressive forces at the repair site with loading could be safely treated with early postoperative weightbearing and unrestricted ROM. Differently from that, a radial tear could be displaced with axial loading dissolving the hoop stress effect, which may require a more conservative approach, with non-weightbearing postoperative strategy with restricted ROM [5].

Currently, there’s no consensus and evidence for a standardized postoperative rehabilitation protocol after meniscal repair. Several variations exist in the literature concerning postoperative weightbearing, range of motion (ROM) and return to sports [6–9].

In order to decrease the rate of muscle atrophy and the development of strength deficits, many authors support the use of accelerated rehabilitation protocols with early weightbearing and unrestricted range of motion after meniscal repair regardless of the tear type [6, 8, 10, 11]. So, considering the paucity of information regarding the ideal protocol, an evidence-based rehabilitation strategy after isolated meniscal repair is needed. A good understanding of the tissue-architecture and biomechanics of the meniscus, in association with a “modern” classification of traumatic meniscal tears, can guide the surgeon to accurately achieve and personalize the ideal post-operative rehabilitation protocol and return to sports (RTS) strategy.

The aim of this current concept review is to look at the evidence of rehabilitation programs and RTS after meniscal repair, focusing on general and specific protocols per type of injury trying to provide guidelines based on the current state of knowledge.

## Methods

A narrative literature review was performed searching PubMed database to identify relevant articles from January 1985 to October 2021 using keywords (meniscus OR meniscal repair) AND (rehabilitation OR physiotherapy OR physical therapy) AND (RTS OR return to sports) AND (biomechanics OR biomechanical). Randomized controlled trials (RCTs), prospective and retrospective cohort studies, case series, systematic reviews, meta-analyses, cadaveric studies and basic science studies were included. The inclusion criteria were as follows: English language studies reporting rehabilitation procedures and RTS after isolated arthroscopic meniscus repair and biomechanical studies on meniscal lesions or meniscal repair. Exclusion criteria were as follows: Non-English studies, articles not related to meniscus repair, studies focusing on results after meniscus transplantation and studies with concomitant procedures (ligament reconstruction, osteotomy, cartilage repair).

## Results

### Biomechanical studies

Generally, the restrictions in ROM and the limitations in weight-bearing, should protect the meniscal repair from increased mechanical load, avoiding compressive overload and shear stresses on the surgical repair [12]. However, biomechanical evidence in few cadaveric studies prompt that high degrees of flexion (90°) and early weight bearing might be safe for particular types of meniscal tear [12–15]. In a porcine cadaveric model, Richards et al. strived to analyze the effects of compressive load in cases of longitudinal vertical and radial meniscus tears [15]. They showed that, in the longitudinal vertical tear scenario, weight-bearing reduced the meniscus and stabilized the repair. Differently, in radial tears, the axial compressive loading might dislocate the lesion instead of reducing it. Furthermore, Becker et al. evaluated meniscofemoral contact pressure while cycling the knee from extension to 90° of flexion after meniscal repair [13]. The study demonstrated that even if the pressure increased in both compartments during flexion, the meniscal repair had no impact. In case of posteromedial meniscal tears, Ganley et al. investigated knee flexion and loading on meniscal healing, using CT scans and special metal markers imbedded in the meniscal lesion [14]. The authors found that at different flexion angles and after 100 lbs of loading the meniscal tear didn’t show any significant gapping. Similarly, Lin et al., in order to assess the effect of postoperative ROM following meniscal repair on a cadaveric model, created a 2.5 cm posteromedial meniscal tear and repaired it with inside-out sutures. Furthermore the authors measured the displacement at high degrees of flexion (90°, 110° and 135°) finding that neither the meniscal tear nor the meniscal repair showed significant gapping [16].

Some authors recommend non-weightbearing and immobilization in full extension, because during flexion the peripheral posterior horn tears move away from the capsule but reduce in extension [17, 18]. However, this finding has not been shown to be beneficial from any clinical point of view [17, 18].

Differently, other investigators proposed immobilization in different degrees of flexion, and others still favor limited early motion protocols [19, 20].

From the biomechanical point of view, postoperative ROM and weight-bearing can affect meniscal healing after repair. Hence, the interaction between tear pattern and knee biomechanics can help the surgeon to properly assess the most suitable postoperative plan.

A modern approach is to tailor an individualized protocol based upon the type of lesion, its location, extent, quality of the repaired tissue and strength of the suture.

Proposed by the authors and supported by biomechanical evidences, in cases of menisco-synovial ramp lesion, hypermobility of the lateral meniscus and a longitudinal vertical tear, the axial compressive load seems to protect and reduce these lesions and an accelerated rehabilitation protocol may be proposed [12–15]. Differently, in case of radial tears scenario, the compressive loading appears to dislocate the lesion instead of reducing it, leading to a restricted rehabilitation program [15].

Despite more aggressive protocols allowing for free ROM and weightbearing as tolerated immediately after meniscal repair, the authors recommend a 90° of flexion as a comfortable restriction, regardless of the type of the lesion [21]. This recommendation is supported by Ahmed et al. The authors demonstrated, using a pressure distribution transducer for in-vitro static measurements in synovial joints, that up to 85% of the load advances through the menisci with the knee in 90° of flexion, while 50% of the load passes through the meniscus in extension [22].

### Accelerated vs Restricted protocols

There is no consensus in the literature regarding the optimal rehabilitation protocol following meniscal repair [23–26]. An accelerated rehabilitation protocol advocates early partial weightbearing and unrestricted range of motion after meniscal repair regardless of the tear type [6, 8, 10, 11]. Differently to that, a restricted program recommends to use limitations in ROM and a more conservative non-weightbearing postoperative course [5].

Only one prospective randomized trial comparing the impact of accelerated regimen versus restricted rehabilitation protocol has been described [21]. Lind et al. involved 60 patients with a diagnosis of vertical meniscal lesion, treated with an all-inside repair technique and randomized by rehabilitation regimens. The accelerated group was allowed to immediately load the knee with weight-bearing as tolerated, associated with immediate ROM from 0° to 90° of flexion. The restricted group needed to wear a hinged brace and gradually increased the ROM up to 90° in association with a touch-down weight bearing for six weeks after the surgery. There were no differences in functional outcome score and healing rate at two years of follow up. Therefore, the authors concluded that free accelerated rehabilitation protocol was safe with a low failure rate.

Following the concept of accelerated protocol based upon the tear’s characteristics, Kocabey et al. reported excellent results for different lesions [27]. For longitudinal tears less than three cm, they proposed weight-bearing as tolerated without using a brace in association with a ROM limited to 0°-125° up to the six weeks. However, for lesions bigger than three cm, patients were wearing a protective locked brace with partial weight bearing with ROM limited to 125° until eight weeks after the surgery. In the complex and radial tears cases, wearing a brace with weight bearing as tolerated with ROM limited to 90° for six to eight weeks were recommended. Furthermore, considering aggressive and accelerated rehabilitation protocols, Mariani et al. showed promising results in 22 patients who underwent outside-in suture in meniscal longitudinal tears of the posterior horn of the medial meniscus. Full weight bearing without ROM restrictions were immediately allowed, and on MRI re-examination only three patients demonstrated signs of re-tear with one mm of gapping [11].

Regarding the efficacy and safety of accelerated protocols in radial tears cases, there are still a lot of concerns. Using a restrictive protocol, two studies reported good results after repair of isolated radial tears of the lateral meniscus [28, 29]. On the other hand, favorable results using a restricted protocol were described by Noyes et al. The authors showed excellent results limiting weight-bearing in association with restricted ROM (120°) over six weeks [30]. Similarly, Logan et al. reported high rate of return to sports (RTS) (81%) using a restricted rehabilitation program with protected weight-bearing and limitation of ROM for six weeks for all types of meniscal lesions [31].

Differences or similarities among the studies previously described are summarized in Table 2.

**Table 2:** Main findings of accelerated and restricted rehabilitations protocols described in literature

Manuscript	No of patients	WB limitations	ROM limitations	Failure rate and follow-up	Level of evidence
Choi et al. [28]	14	Toe-touch WB for 6 weeks, followed by a gradual increase of weight- bearing over the following 4 weeks	ROM exercises were allowed from 0° to 90° of flexion for 6 weeks	Failure rate 7% Follow-up 36 months	Case series: Level of evidence 4
Haklar et al. [29]	5	No WB 6–8 weeks	ROM 0°-120°	Failure rate 0% Follow-up 31 months	Non-randomised cohort: Level of evidence 3
Kocabey et al. [27]	52	Immediate WB as tolerated	ROM 0°-125°	Failure rate 4% Follow-up 10 months	Retrospective case series: Level of evidence 4
Lind et al. [21]	60 (32 accelerated protocol, 28 restricted protocol)	Accelerated protocol: 2 weeks toe-touch WB Restricted protocol: 6 weeks toe-touch WB	Accelerated protocol: ROM 0° − 90°, without brace, then return to normal activities Restricted protocol: 6 weeks with locked brace, gradual increase ROM to 90°	Failure rate 28% (accelerated) 36% (restricted) Follow-up 24 months	Randomised controlled clinical trial: Level of evidence 1
Logan et al. [31]	42	Protected WB for 6 weeks	ROM 0°-120° for 6 weeks	Failure rate 24% Follow-up 102 months	Case series: Level of evidence 4
Mariani et al. [11]	22	Immediate WB as tolerated	Immobilisation with brace locked in full extension for 1 month, passive ROM 0° − 90° for 2 weeks, than gradual increase	Failure rate 9% Follow-up 28 months	Non-randomised cohort study: Level of evidence 3
Noyes et al. [30]	29	Partial WB for 4 or 6 weeks	ROM 0°- 135° for 6 weeks	Failure rate 25% Follow-up 51 months	Non-randomised cohort study: Level of evidence 3

*WB* Weightbearing, *ROM* Range of motion

Following these biomechanical premises, keeping in mind the macro-geometry and tissue-architecture of circumferential and radial fibers of the menisci and considering the studies described in the literature (Table 2), a tailored and customized rehabilitation protocol after meniscal repair can be proposed. According to the integrity of the circumferential fibers, the senior author described and classified two different types of meniscal lesions: stable and unstable.

In longitudinal (oblique, vertical) lesions, the biomechanics of the circumferential fibers are preserved. Consequently, the hoop-stress effect is maintained making these tears types in general more stable [4]. Differently to that, complete radial tears should be treated more conservatively, because the circumferential hoop fibers are disrupted [4]. Proposed approaches for each category of lesions are summarized in Table 3.

**Table 3:** Approaches for each category of meniscal lesions

Meniscal Repair	ROM	Weight Bearing	Strengthening Exercises
Longitudinal Tear	0–90°	Partial (20 kg) WB 4 weeks	3 months after surgery
Ramp Lesion	0–90°	Partial (20 kg) WB 4 weeks	3 months after surgery
Hypermobile Lateral Meniscus	0–90°	Partial (20 kg) WB 4 weeks	3 months after surgery
Root Lesion	0–90°	No WB 6 weeks	4–5 months after surgery
Radial Tear	0–90°	No WB 6 weeks	4–5 months after surgery

*WB* Weightbearing, *ROM* Range of motion

In the cases of menisco-synovial ramp repair, hypermobility of the lateral meniscus fixation and longitudinal tear suture, where the hoop tensile stress effect is preserved, partial weight bearing (20 kg) is allowed for the first four weeks after surgery, with weight bearing as tolerated for the following weeks [11, 30]. Range of motion is limited to 90° of flexion using a protective brace for six weeks [28].After six weeks, progression beyond 90° of flexion is allowed, but knee loading in deep flexion is limited for four months postoperatively [30]. Strengthening exercises with weights starts after three months and isolated strengthening of hamstrings does not usually start before four months [32].

Concerning root and radial repairs, where circumferential fibers are disrupted, no weight bearing is allowed for the first six weeks after the surgery [28, 29].Then, progressive return to full weight bearing is allowed. Finally, the protocol is the same as aforementioned, but strengthening is delayed by one month [32].

### Return to sport (RTS)

Meniscal repair is largely performed in young and active patients including elite and professional athletes, but only a few studies looked at the RTS after meniscal repairs using a sport-specific approach [33–35]. Only six studies specifically evaluated RTS in either mixed-level or professional athletes after isolated meniscal suture [31, 36–40]. No biomechanical studies evaluating the RTS rates after meniscal repair were found. However, a better understanding of RTS parameters could guide and help the surgeons while aiding discussion about recovery after meniscal repair with athletes and team physicians. Eberbach et al., in a recent systematic review, investigated sport-specific outcomes, RTS and failure rate after meniscal repair, evaluating 28 general studies [33]. The authors showed that repairing a meniscal tear is correlated to good sport-specific outcomes and RTS in either recreational and professional athletes. 89% of all the patients were capable of RTS to their preinjury activity level, with a slight difference between mixed-level populations (90%) and professional athletes (86%). Concerning the level of activity, the Tegner rating scale was used in 17 studies. Regarding preoperative and postoperative levels, the Tegner score improved from 3.5 ± 0.3 to 6.2 ± 0.8. On the other hand, comparing preinjury and postoperative levels, a small decrease of the Tegner score from 6.3 ± 1.1 to 5.7 ± 0.8 was reported, without showing any clinical impairment. Finally, a global failure rate of 21% was shown, with a lower level in professional athletes (9%) compared to the recreational group (22%) [33].

Differences or similarities among the studies specifically evaluating RTS are summarized in Table 4.

**Table 4:** Studies analyzing RTS after isolated meniscal repair

Manuscript	No of patients	RTS (%)	Time to RTS (median)	Meniscal tears
Alvarez-Diaz et al. [36]	14	92	4.3 months	Longitudinal vertical
Logan et al. [31]	7	71	5.6 months	Longitudinal vertical (82.2%), Complex (11.1%), Partial (6.7%)
Griffin et al. [37]	16	75	4.3 months	Longitudinal vertical (63%) Bucket handle (27%)
Pujol et al. [38]	21	95	10 months (same level)	Longitudinal horizontal
Tucciarone et al. [39]	20	90	-	Longitudinal vertical (90%) Bucket handle (10%)
Vanderhave et al. [40]	14	100	6.5 months	Longitudinal vertical (32%), bucket-handle (31%), and complex (37%)

*IKDC* International Knee Documentation Score, *KOOS* Knee injury and Osteoarthritis, *RTS* Return to sport

Several factors may influence healing after meniscal repair, delaying RTS activity. These factors include tear type, medial versus lateral meniscus tear, and the presence of concomitant injuries. Lyman et al. showed risk factors for meniscectomy after meniscal suture, analyzing 9609 outpatient meniscal repairs collected from a statewide database of all ambulatory surgery in New York. The authors identified patients aged 40 years and older, injuries of the lateral meniscus and meniscal suture associated with a concomitant ACL reconstruction as protective factors after meniscal repair [41]. However, concerning isolated medial versus lateral meniscus repair, the data shown in literature are conflicting. Tuckman et al., differently to Lyman and colleagues, revealed a failure rate of 20% for the medial meniscus compared with 44% in the lateral meniscus [42]. Regardless of the meniscal tear type, almost 90% of the patients are able to return to sports after isolated meniscal repair [33].

As already highlighted for rehabilitation, taking into consideration the tissue-architecture of circumferential and radial fibers of the menisci, and considering the studies described in the literature (Table 3), a tailored and customized RTS protocol after isolated meniscal repair should be based upon the tear type, the meniscus stability, and the type of sports, which can be classified according to the Tegner Activity Scale [43]. Since there are no studies in the literature evaluating the RTS (rate/time) after meniscal repair in a longitudinal horizontal or vertical tears, RTS for competitive and contact sports activity is advised after four to six months [31, 36–40].

In the case of radial or root tear scenario and due to the instability of the lesion itself, the senior author suggest delaying RTS until nine months in professional contact and pivoting sports.

For an unstable lesion in a professional contact and pivoting sports athlete, the Authors recommend a RTS three months later compared to the stable tear.

**Fig. 2:**
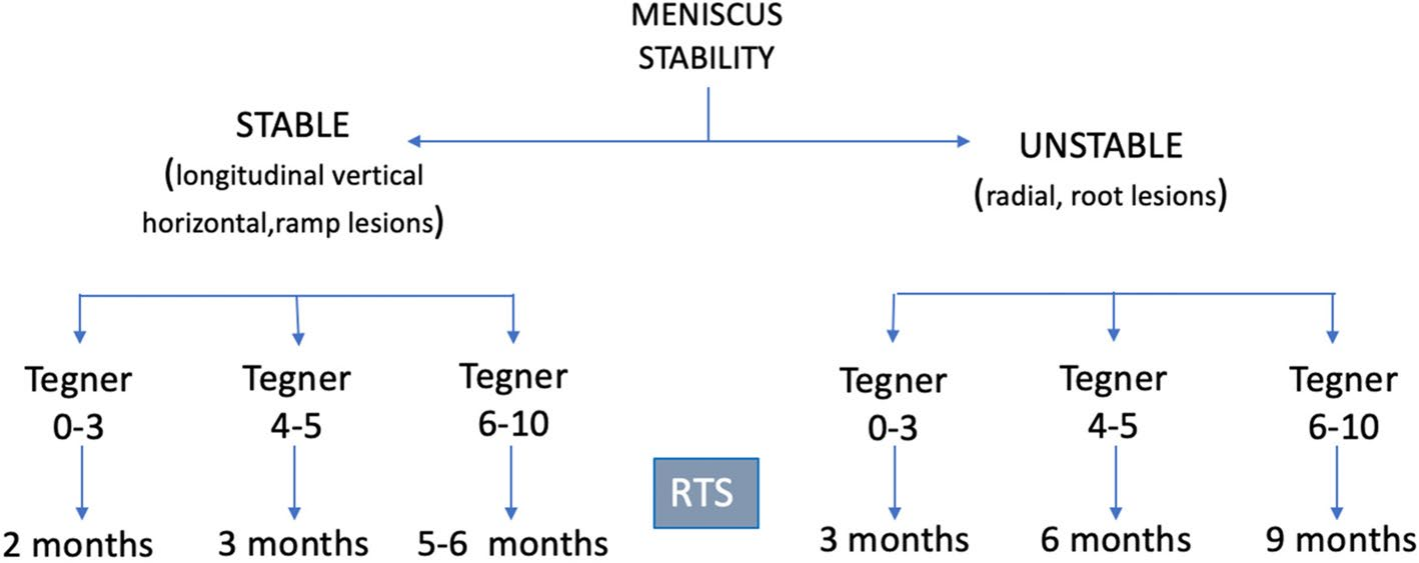
A RTS protocol after isolated meniscal repair is developed and proposed by the authors in accordance with the Tegner Activity Scale and meniscus stability

The “too much too soon” return to high activity is a risk factor for treatment failures, especially in a younger population [44]. General approaches for each category of tears are summarized in Fig. 2.

## Conclusion

In the current literature there is a lack of consensus on the optimal post-operative rehabilitation and the best RTS program. However, biomechanical evidence suggests that tailoring an individualized protocol based upon the type of lesion and its stability can be reasonable. When the hoop tensile stress effect is preserved, an accelerated rehabilitation program may be suggested. In contrast, when circumferential hoop fibers are disrupted, a restricted rehabilitation protocol may be recommended.

Additional biomechanical and more RCT studies are needed to improve our knowledge and reach a consensus on the ideal rehabilitation protocol after meniscal repair.
